# Nature–Inspired Metaheuristic Optimization for Control Tuning of Complex Systems

**DOI:** 10.3390/biomimetics10010013

**Published:** 2024-12-30

**Authors:** Jesús Garicano-Mena, Matilde Santos

**Affiliations:** 1 ETSI Aeronáutica y del Espacio—Universidad Politécnica de Madrid, 28040 Madrid, Spain; 2 Center for Computational Simulation (CCS), 28660 Boadilla del Monte, Spain; 3 Institute of Knowledge Technology, Complutense University of Madrid, 28040 Madrid, Spain

**Keywords:** Metaheuristics Algorithm, Whale Optimization Algorithm (WOA), Antlion Optimization Algorithm (ALO), complex dynamics systems, Hoop & Ball electromechanical system, wind energy conversion system

## Abstract

In this contribution, a methodology for the optimal tuning of controllers of complex systems based on meta–heuristic techniques is proposed. Two bio-inspired meta-heuristic optimization algorithms –the Antlion Optimizer (ALO) and the Whale Optimization Algorithm (WOA)– have been applied to two different dynamic systems: the Hoop & Ball electromechanical system, a system where a linearized description is adequate; and to a Wind Turbine–Generator–Rectifier, as an example of a complex non-linear dynamic system. The performance of the ALO and WOA techniques for the tuning of conventional PID controllers is evaluated in relation to the number of agents $n_{S}$ and the maximum number of iterations $n_{MaxIter}$; given the stochastic nature of both methods, repeatability is also addressed. Finally, the computational effort required for their implementation is considered. By analyzing the obtained metrics, it is observed that both methods provide comparable results for the two systems considered and, therefore, the ALO and WOA techniques can complement each other by exploiting the advantages of each of them in controller tuning.

## 1. Introduction

Most real systems, especially in the industrial and engineering fields, do not require very sophisticated control strategies to achieve good performance. However, to achieve the required specifications of precision, minimum control effort, energy savings, etc., it is essential that the regulators are well designed and configured [1].

The task of adjusting regulators is a laborious and non-trivial job. Hence, numerous techniques have been applied for the optimal adjustment of their parameters, and it remains an open topic for research since the requirements are increasingly demanding [2].

Furthermore, thanks to technological and computational advances, it has been possible to address the control of increasingly complex, non-linear systems, with coupled variables and disturbances. But this requires a greater effort in the design and adjustment of the controllers.

In this work, this challenge is addressed by applying two meta-heuristic techniques for the optimal adjustment of a conventional controller for two real systems.

The conventional controller is a Proportional–Integral–Derivative (or *PID*), which is possibly the most widespread feedback control strategy in industry [3]. The *PID* control approach offers, in its most simplified setting [4], a control action u(t) determined as the weighted superposition of an error measure $e\left(t\right)\equiv y_{ref}\left(t\right)-y\left(t\right)$, its temporal integral and its derivative, namely:

(1) $$\begin{array}{c} u\left(t\right)=K_{p}e\left(t\right)+T_{i}\int e(\tau )d\tau +T_{d}\frac{de(t)}{dt}. \end{array}$$

This simple formulation is sufficiently flexible to, upon proper setting (i.e., *tuning*) of the parameters $\left(K_{p},\;T_{i},\;T_{d}\right)$, provide satisfactory results in many situations. Note that, on many occasions, there might be many parameter combinations that lead to solutions of similar merit [5].

There are many strategies to find those parameters $\left(K_{p},T_{i},T_{d}\right)$, e.g., the now classical Ziegler–Nichols heuristic method, or the pole placement approach, for instance; these methods work specially well for systems that can be accurately described with low order models, and wherever noise is not a concern [6].

More recently, more sophisticated, *intelligent* methods have been introduced, and have been applied not only to tackle new control challenges, but also to revisit the *PID* tuning problem [7,8,9]. Among these intelligent techniques, the so–called metaheuristic optimization algorithms have risen as a promising strategy [10]. Genetic Algorithms (*GA*) and Particle Swarm Optimization (*PSO*) algorithms are well known methods of this class, that happen to be inspired by biological processes [11,12,13,14]. In this sense, neither *GA* nor *PSO* are a novelty: a plethora of nature–inspired metaheuristic optimization algorithms exists; a taxonomy for those is provided in [8].

Among those methods, in this work we focus on two recent and relatively more unknown nature–inspired metaheuristic optimization algorithms: the Antlion Optimizer (or ALO [15]), and the Whale Optimization Algorithm (or WOA [16]). The first one draws from the stalking behavior of antlions, whereas the second tries to mimick the cooperative hunting behavior of humpback and Bryde’s whales. Both methods evidence an effective performance on a wide array of non–linear optimization problems.

Specifically, this paper analyzes the performance of these two bio–inspired metaheuristic optimization algorithms for tuning proportional–integral controllers (*PI*, i.e., with $T_{d}=0$ in Equation (1)). The objective is to see their applicability in the control of complex non–linear systems and their dependence on the algorithms initial and configuration parameters. To do so, their application to two different dynamic systems is compared: the electromechanical hoop & ball system (HB), as a system where a linearized description is adequate; and a wind turbine–generator–rectifier–load (WTGRL) system, as an example of a system with a complex and nonlinear dynamics. To our knowledge, these techniques have not been applied in the field of control of these systems. Our objective is to evaluate the applicability of the ALO and/or WOA techniques to efficiently tune the *PI* controllers using only information of feasible ranges for $K_{p}$ and $T_{i}$ gains.

The main contributions of this paper can be summarized as:

- a methodology is proposed for the automatic optimal tuning of conventional *PID*  controllers;
- this methodology uses two advanced metaheuristic optimization algorithms of biological inspiration, the techniques ALO and WOA;
- the only information needed is the feasible ranges for $K_{p}$ and $T_{i}$;
- the performance of both optimization techniques has been compared in two nonlinear dynamic systems of increasing complexity, the hoop and ball system and the wind turbine-generator-rectifier-load system;
- they have been compared with the solutions provided by other evolutionary techniques for the same hoop and ball system found in the literature;
- a sensitivity analysis of the main parameters of the algorithms has been performed in terms of goodness of solution and computational time.

The rest of the document is organized as follows: next, we present the Methodology used in this work, including the systems modeling and the ALO & WOA optimization techniques description. In Section 3, the results obtained with the two metaheuristic algorithms applied to the two non–linear systems are discussed. Finally, in Section 4, we present the conclusions that can be drawn from this study, together with some recommendations for using the ALO and WOA to sintonize *PID* controllers, and future works.

## 2. Methodology

In Section 2.1 and Section 2.2, we present the models of the dynamic systems considered in this work. Next, in Section 2.3, we briefly review the two bioinspired metaheuristic algorithms leveraged for controller tuning.

### 2.1. The Hoop & Ball System

**Figure 1:**
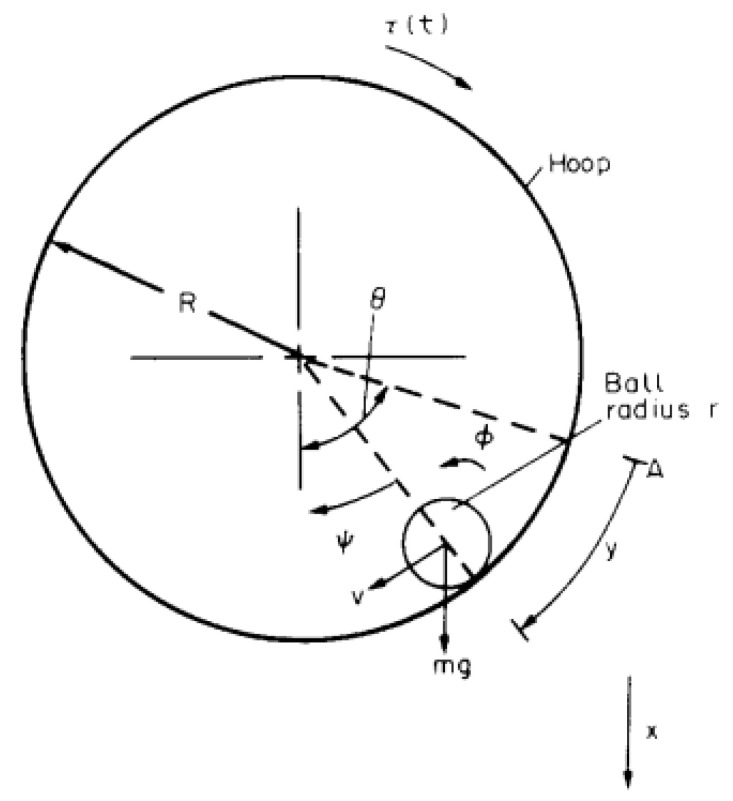
HB system: sketch, reproduced from [17].

The Hoop & Ball (HB) system, initially presented in [17], is a electro–mechanical non-linear dynamic system for which a linearized description is adequate over a relative wide range of conditions. This system consists of a small spherical bill of radius $r_{b}$, which is constrained to roll along the grooved interior surface of a hoop of radius $R>r_{b}$. This hoop is actioned by a servomotor connected to the hoop principal axis. A sketch of the HB system is shown in Figure 1.

This system is well established as an academic benchmark for testing control solutions. Although simple in construction, it shows an interesting behavior. Moreover, the ball–hoop system offers a useful analogy of the sloshing phenomenon, which is the fluid–structure interaction happening when a deposit partially filled with a liquid experiences a sudden acceleration/deceleration, i.e., in road or ship transportation, and more recently, it has been also considered for liquid metal cooled nuclear reactors.

Mathematical models of the Hoop–Ball systems of varying complexity are available in the bibliography [17,18]. The most general model is this non–linear system of equations [19]:

(2) $$\begin{array}{ccc} \left[I_{a}+m{\left(R-r\right)}^{2}\right]\ddot{\theta }+b_{m}\dot{\theta }-m\frac{{\left(R-r\right)}^{2}}{R}\ddot{y} & = & -mg\left(R-r\right)\sin\left(\psi \right)+\tau \left(t\right),\\ \left[I_{b}+m\frac{{\left(R-r\right)}^{2}}{R}\right]\ddot{y}+\frac{b_{b}}{r^{2}}\dot{y}-m\frac{{\left(R-r\right)}^{2}}{R}\ddot{\theta } & = & mg\frac{R-r}{R}\sin\left(\psi \right), \end{array}$$

where *R* and $r_{b}$ are respectively the hoop and ball radii, whereas $r<r_{b}$ is the ball rolling radius; and *m* is the ball mass. The inertia moments of hoop and ball are $I_{a}=\frac{1}{2}M\;R^{2}$ and $I_{b}=\frac{2}{5}M\;r_{b}^{2}$. A servomotor actions the hoop with a moment τ(t); this servomotor is affected by a friction $b_{m}$, whereas the rolling resistance of the ball is $b_{b}$. Finally, the relationship $\psi =\theta -\frac{y}{R}$ closes the system of equations.

The system (2) can be regard as a first order non–linear system by defining $x_{1}\equiv \theta \left(t\right)$ and $x_{3}\equiv y\left(t\right)$, so that $\dot{\theta }={\dot{x}}_{1}\equiv x_{2}$ and $\dot{y}={\dot{x}}_{3}\equiv x_{4}$. In this way, we obtain

(3) $$\begin{array}{ccc} A_{\theta }{\dot{x}}_{2}+B_{\theta }x_{2}+C{\dot{x}}_{4} & = & D_{\theta }\sin\left(\theta -\frac{y}{R}\right)+\tau \left(t\right),\\ C{\dot{x}}_{2}+B_{y}x_{4}+A_{y}{\dot{x}}_{4} & = & D_{y}\sin\left(\theta -\frac{y}{R}\right), \end{array}$$

Introducing now $x\left(t\right)\equiv {\left[x_{1}\left(t\right),\;x_{2}\left(t\right),\;x_{3}\left(t\right),\;x_{4}\left(t\right)\right]}^{T}$, it is possible to reformulate Equation (3) as:

(4) $$\begin{array}{c} \underset{M_{0}}{\underbrace{\left[\begin{array}{cccc} 1 & 0 & 0 & 0\\ 0 & A_{\theta } & 0 & C\\ 0 & 0 & 1 & 0\\ 0 & C & 0 & A_{y} \end{array}\right]}}\frac{d}{dt}x\left(t\right)=\underset{A_{0}}{\underbrace{\left[\begin{array}{cccc} 0 & 1 & 0 & 0\\ 0 & -B_{\theta } & 0 & 0\\ 0 & 0 & 0 & 1\\ 0 & 0 & 0 & -B_{y} \end{array}\right]}}x\left(t\right)+\underset{\mathrm{NL}}{\underbrace{\left[\begin{array}{c} 0\\ D_{\theta }\\ 0\\ D_{y} \end{array}\right]\sin\left(\theta -\frac{y}{R}\right)}}+\underset{F_{0}}{\underbrace{\left[\begin{array}{c} 0\\ \tau \\ 0\\ 0 \end{array}\right]}}, \end{array}$$

It can be also expressed in more compact form as:

(5) $$\begin{array}{c} \frac{d\;}{dt}x\left(t\right)=M_{0}^{-1}\;A_{0}\;x\left(t\right)+M_{0}^{-1}\;\mathrm{NL}+M_{0}^{-1}\;F_{0}. \end{array}$$

In this expression, it is possible to identify, on the right side, a linear contribution, another non–linear contribution proportional to $\sin\left(\theta -\frac{y}{R}\right)$, and the forcing term.

Under the assumption that r≪R and ψ≪1, $\sin\left(\theta -\frac{y}{R}\right)∼\theta -\frac{y}{R}$. Thus, in the linearized model, the servomotor would act directly the hoop, which in turn excites the ball movement. The specific parameters of the HB model considered in this work are: g=9.81 m/s^2^, R=0.085 m, M=0.10 kg, m=0.02812 kg, r=0.0095 m, $r_{b}=0.009525\;$ m, $b_{m}=0.1\;$ kg m^2^/s and $b_{b}=3.22\times 10^{-6}\;$ kg m^3^/s.

**Figure 2:**
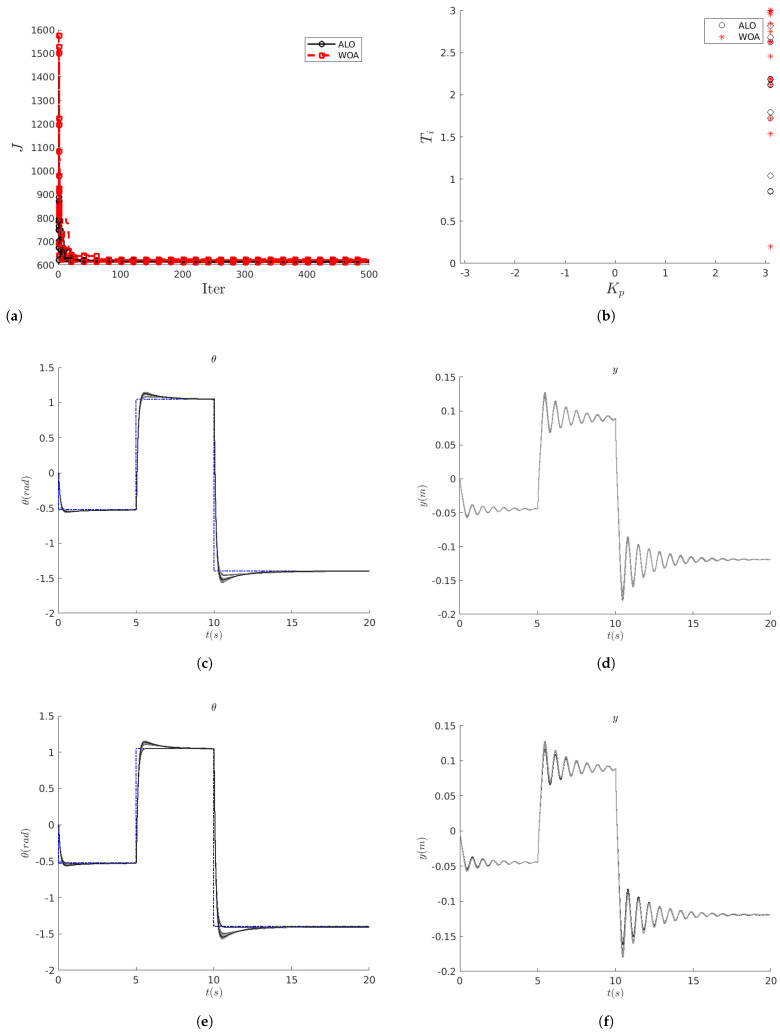
HB system: in (**a**), evolution of the cost function; in (**b**), different solutions found by the optimization algorithms ALO (∘) and WOA (*); in (**c**,**d**), variability of the system solutions identified by ALO; in (**e**,**f**) the same for WOA.

In order to discretize the HB model, the matlab command ode45 was used. The hoop axis is controlled by a Proportional Integral (*PI*) controller, where the control signal is generated with feedback of the signal θ:

(6) $$\begin{array}{c} u\left(t\right)=K_{p}\left(\theta \left(t\right)-\theta_{ref}\left(t\right)\right)+T_{i}\int \left(\theta \left(\tau \right)-\theta_{ref}\left(\tau \right)\right)d\tau , \end{array}$$

where $\theta_{ref}\left(t\right)$ is a sequence of step signals defined for t∈[0,20]s (see e.g., Figure 2c).

The tuning of the $K_{p}$ and $T_{i}$ gain is going to be carried out using the ALO and WOA optimization techniques, with the aim of minimizing the Squared Error norm ${∥\theta \left(t\right)-\theta_{ref}\left(t\right)∥}_{2}^{2}$. The search space is defined for $|K_{p}|<3.1$ and $T_{i}\in \left[0,\;3\right]$ [20].

### 2.2. The Wind Turbine–Generator–Rectifier–Load System

Wind energy conversion systems are a typical example of a complex system, involving many mechanical, hydraulic, electrical, and electronic (possibly combined) subsystems that interact to reach a common goal, i.e.,extract power from the momentum in a wind stream [21]. Therefore, modeling such systems can easily become complicated [22,23]. Regarding the goals of this contribution—extracting useful knowledge from the comparison of the ALO and WOA algorithms in the context of intelligent control of complex systems—we consider a relatively simple yet sufficiently rich model, inspired in those discussed in [24,25,26,27].

In this wind turbine–generator–rectifier–load system, a 12.3 kW three–bladed horizontal axis wind turbine is considered. It drives directly (i.e., no gearbox) a three–phase salient pole permanent magnet synchronous electric generator. This generator excites a rectifier module that enacts the AC/DC conversion; the rectifier, in turn, feeds through a DC-DC boost converter a resistive load of $R_{L}=30\;\Omega$.

**Figure 3:**
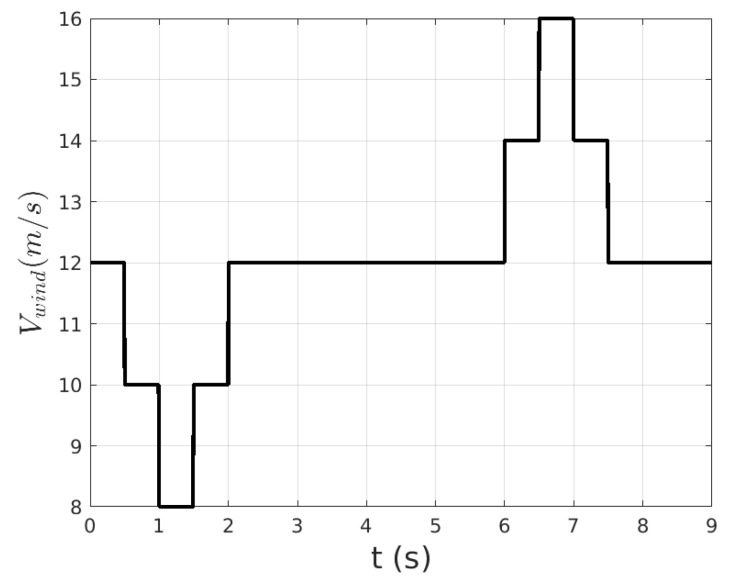
WTGRL system: wind gusts affecting the wind turbine subsystem.

We consider that the turbine is exposed to several wind gusts, Figure 3, acting in the interval t∈[0,9]s. The rectifier subsystem follows a Maximum Power Point Tracking (MPPT) strategy, whereas in the operating regions of rated wind speed, a Proportional–Integral (*PI*) controller adjusts the blade angle of attack (β). The angle of attack is constrained to remain in the range $\beta \in [0^{\circ },15^{\circ }]$, and is also affected by a Rate Limiter.

In our model we use powergui: the simulation is performed in discrete mode ($T_{s}=20\times 10^{-6}$ s or $f_{s}=50\;$ kHz), so that the *z*-transfer function of the *PI* controller is:

(7) $$\begin{array}{c} C\left(z\right)=K_{p}+T_{s}\;T_{i}\frac{1}{z-1}. \end{array}$$

**Figure 4:**
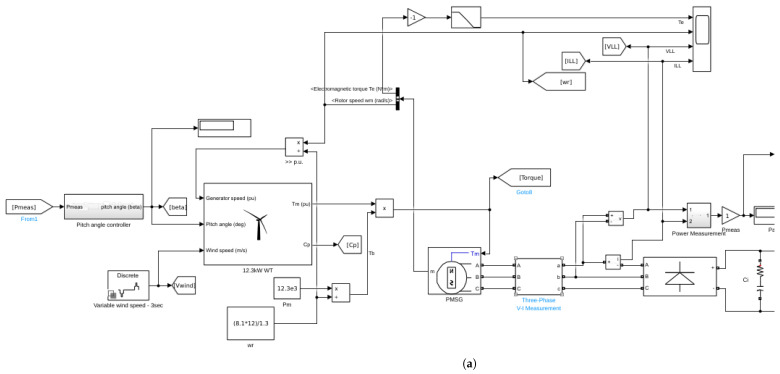

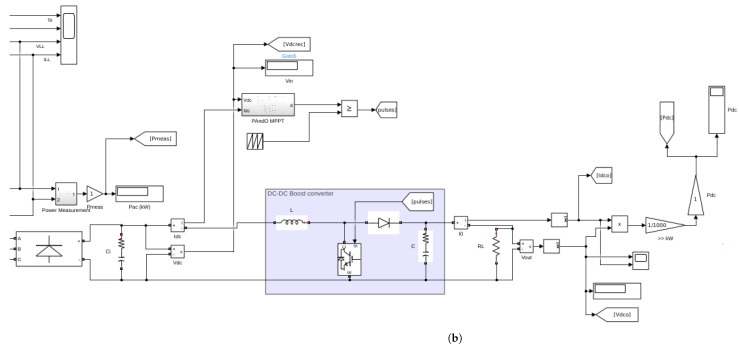
WTGRL system: simulink model of the wind turbine—generator—load with *PI* control for the angle of attack β and MPPT control for the rectifier subsystem. The wind turbine, the generator and the rectifier subsystems are shown in (**a**); the rectifier and the DC-DC boost converter are shown in (**b**).

The simulink model for the WTGRL system is shown in Figure 4.

The tuning values of $K_{p}$ and $T_{i}$ will be obtained with the two nature–inspired metaheuristic optimization algorithms (ALO and WOA). The optimization goal, in this case, is to dissipate as much power at the load $R_{L}$ as possible. This power is given by,

(8) $$\begin{array}{c} P_{DC,0}=V_{DC,0}\;I_{DC,0}. \end{array}$$

Thus, the fitness function *J* is defined as:

(9) $$\begin{array}{c} J=-\sum_{l=1}^{n_{timeSteps}}{\left(P_{DC,0}\left(t=t_{l}\right)\right)}^{2}, \end{array}$$

which is proportional to the discretized integral, quantifying the power dissipated at the load $\int_{0}^{9}{\left(P_{DC,0}\left(\tau \right)\right)}^{2}d\;\tau$. We shall explore the search space limited by $|K_{p}|<5000$ and $T_{i}\in \left[0,\;60,000\right]$ [26,27].

### 2.3. Metahuristic Optimization Algorithms

In this work we consider two metaheuristic optimization algorithms, the *Antlion Optimization Algorithm* (ALO) and the *Whale Optimization Algorithm* (WOA), both coined by Prof. S. Mirjalili’s research group. The biological phenomena that inspire these algorithms are illustrated in [28] and [29], respectively.

As metaheuristic algorithms, ALO and WOA present these advantages:

i they are formulated on simple principles and allow for an easy implementation;
ii the computing of the objective function gradient is not necessary;
iii they can avoid eventual local optima; and
iv they can be applied in many diverse fields.

The implementation of either algorithm is publicly available; and since they share a common interface, so once a script is coded for one of the techniques, it can be easily reused with ay other similar algorithm.

#### 2.3.1. The Antlion Optimization Algorithm

We provide here a brief description of the ALO technique, initially presented in [15]; the corresponding pseudo–code is reproduced in Algorithm 1 [30]. As a flock/swarm type algorithm, ALO employs $n_{S}$ ant agents, but also $n_{S}$ antlion agents within a *d*-dimensional space. The ants move through the space following a random walk in all their coordinates. On the other hand, antlions build *sand pit traps*, whose sizes are larger the lower the value of the objective function (i.e., fitness) at a specific location. The movement of the ants must respect the constraints set by the lower $b_{l}$ and upper $b_{h}$ coordinate vectors. This can be achieved with a random walk given by:

(10) $$\begin{array}{c} A^{k}=\left[0,\;cumsum\left(2\;r\left(t_{1}\right)-1\right),\ldots \;cumsum\left(2\;r\left(t_{IterMax}\right)-1\right)\right], \end{array}$$

if the function r(t) is defined in terms of a uniform random variable Z∼U[0,1], so that r(t)=1 if z∈(0.5,1] and 0 elsewhere. We ensure that all the agents remain in the search space $\left[b_{l},\;b_{h}\right]$ by resorting to the normalization:

(11) $$\begin{array}{c} A_{i}^{k}=c_{i}^{k}+\frac{d_{i}^{k}-c_{i}^{k}}{b_{i}-a_{i}}\left(A_{i}^{k}-a_{i}\right), \end{array}$$

where $a_{i}$ and $b_{i}$ are the minimum and maximum of the random walk for variable *i*, and $c_{i}^{k}$ y $d_{i}^{k}$ are, respectively, the minimum and the maximum of the *i*-th variable at the *k*-th step.

The ALO algorithm starts by randomly distributing the $n_{s}$ ants and the $n_{s}$ antlions over the feasible solution space. Next, the best antlion ${\mathrm{AL}}^{*}$ is identified, namely, the antlion that $\min_{i}\;f\left({\mathrm{AL}}_{i}\left(k=0\right)\right)$. Then, for the number of iterations chosen a priori, $n_{MaxIter}$, the ants wander throughout the search space, whereas the antlions attempt to hunt them down. This guarantees the exploration of the search space. On the other hand, the exploitation of the areas of interest is guaranteed by the progressive contraction of antlion sand pit traps, which can be modeled as:

(12) $$\begin{array}{c} c^{k}=\frac{c^{k}}{I},yd^{k}=\frac{d^{k}}{I}, \end{array}$$

where *I* is a compression ratio, and $c^{k}$/$d^{k}$ are the minimum/maximum of all the variables in the *k*-th iteration.

Note how the combination of the ant random walks with the roulette wheel selection of the antlions contributes to avoid, with high probability, stagnating into local minima.

Even more, the random walk of each ant along every dimension fosters the diversity of the agents involved. And since the sand pit traps relocate to the position of the best ant found during the optimization, the promising areas of the search space are preserved.

Finally, elitism is applied, since the best antlion at every iteration is stored and compared against the best antlion so far (the elite). Algorithmically, ants roam randomly both around the antlion chosen by roulette wheel selection but also around the elite antlion:

(13) $$\begin{array}{c} A_{i}^{k}=\frac{R_{A}^{k}+R_{E}^{k}}{2}, \end{array}$$

where $R_{A}^{k}$ denotes the trajectory around the antlion active in the current iteration, whereas $R_{E}^{k}$ is the trajectory around the elite antlion.

The detailed procedure described above can be formalized in the successive application of three operators that can be found in [15].
**Algorithm 1** The Antlion Optimization Algorithm [15].  1:**Algoritmo** ALO($n_{S},\;n_{MaxIter},\;b_{l},\;b_{h},\;d,\;f$)  2:    Random initialization of ants ${\left\{A_{i}\right\}}_{i=1}^{n_{S}}$ and antlions ${\left\{{\mathrm{AL}}_{i}\right\}}_{i=1}^{n_{S}}$.  3:    Calculate the fitness of ants, $M_{O,A}\left(X_{i}\right)$ and antlions $M_{O,AL}\left(X_{i}\right)$.  4:    Identify the best antlion ${\mathrm{AL}}^{*}$ (*elite*).  5:    **while** $k\leq n_{MaxIter}$ **do**  6:        **for** $i=1,\;\ldots ,\;n_{S}$ **do**  7:           Choose an antlion using *roulette wheel*.  8:           Update *c* and *d* with Equation (12).  9:           Create a random walk (Equation (10)) and normalize it (Equation (11)).10:           Update the *i*-th ant position (Equation (13)).11:        **end for**12:        Recalculate the fitness of every ant.13:        Replace an antlion with the corresponding ant, provided it becomes fitter.14:        Update the elite agent if another antlion becomes better.15:    **end while**16:    **return** elite17:**end Algoritmo**

#### 2.3.2. The Whale Optimization Algorithm

A brief description of the WOA technique with the corresponding pseudo–code is reproduced here as in Algorithm 2 and [31]. WOA considers a pod (or herd) of $n_{S}$ whales (or agents or particles), that evolve throughout a *d*-dimensional space in search of prey, i.e., the local and/or global minima of the function f to be optimized. The whales movement remains in the interior of the cartesian product defined by the corresponding components of the lower $b_{l}$ and upper $b_{h}$ constrain vectors.

The algorithm begins by randomly distributing the $n_{s}$ whales over the feasible solution space; then, the best solution so far, $X^{*}$, is singled out, namely, that one fulfilling $\min_{i}\;f\left(X_{i}\left(k=0\right)\right)$.

For a pre-set number of iterations $n_{MaxIter}$, the whale positions are updated, alternating phases of:

i prey search (exploration or diversification),
ii prey encircling, and
iii bubble net attacking (exploitation or intensification).

At every iteration *k*, and for every whale, a random number $p_{i}\left(k\right)∼U\left[0,\;1\right]$ is considered. If $p_{i}\left(k\right)<0.5$, and depending on the magnitude of a vector A, the method alternates between the circling phase –if $\left|A\right|<1$– or the search of prey phase –when $\left|A\right|\geq 1$.

The circling phase is described by equations:

(14) $$\begin{array}{c} D_{i}=\left|C_{i}⊙X^{*}\left(k\right)-X_{i}\left(k\right)\right|,\\ X_{i}\left(k+1\right)=X^{*}\left(k\right)-A_{i}⊙D_{i}, \end{array}$$

where A=2a⊙r−a y C=2r, with r is a vector with random components, and a is a vector whose components shrink linearly with *k* from 2 to 0. These equations describe the global effect of having the best–positioned whale so far (i.e., with a lower f) to *warn* her mates of how promising her current location is.

The exploratory phase, namely the search of preys, is controlled by equation:

(15) $$X_{i}\left(k+1\right)=X_{rand}-A_{i}⊙D_{i},$$

but here $D_{i}$ is given by:

(16) $$D_{i}=\left|C_{i}⊙X_{rand}\left(k\right)-X_{i}\left(k\right)\right|.$$

On the other side, whenever $p_{i}\left(k\right)\geq 0.5$, the hunting phase is active, and the agents position is updated according to:

(17) $$\begin{array}{c} X_{i}\left(k+1\right)=\left\{\begin{array}{c} X^{*}\left(k\right)-A_{i}⊙D_{i},\mathrm{si}q_{i}\left(k\right)<0.5\\ \left|X^{*}\left(k\right)-X_{i}\left(k\right)\right|\;e^{b\;l}\cos\left(2\;\pi \;l\right)+X_{rand},\mathrm{si}q_{i}\left(k\right)\geq 0.5, \end{array}\right. \end{array}$$

where yet another random number $q_{i}\left(k\right)∼U\left[0,\;1\right]$ controls whether the whale encircles her prey, or whether she *decides* to approach the prey describing the famous spiral of decreasing radius.

**Algorithm 2** Whale Optimization Algorithm [16].
  1:**Algoritmo** WOA($n_{S},\;n_{MaxIter},\;b_{l},\;b_{h},\;d,\;f$)  2:    Random whale positions initialization ${\left\{X_{i}\right\}}_{i=1}^{n_{S}}$.  3:    Calculate the fitness of every whale, $f(X_{i})$.  4:    Identify the best whale $X^{*}$.  5:    **while** $k\leq n_{MaxIter}$ **do**  6:        **for** $i=1,\;\ldots ,\;n_{S}$ **do**  7:           Update a, A, C, *l*, *p* y *q*.  8:           **if** p<0.5 **then**  9:               **if** $\left|A\right|<1$ **then**10:                   Use Equation (14).11:               **else if** $\left|A\right|\geq 1$ **then**12:                   Choose a whale at random, $X_{rand}\left(k\right)$, and use Equation (15).13:               **end if**14:           **else**15:               Use Equation (17).16:           **end if**17:        **end for**18:        Identify whales that wandered beyond region $\left[b_{l},\;b_{h}\right]$, and *bring them back*.19:        Recalculate the fitness for every whale, $f(X_{i})$.20:        Update $X^{*}$ if a better solution was found.21:    **end while**22:
**end Algoritmo**



**Figure 5:**
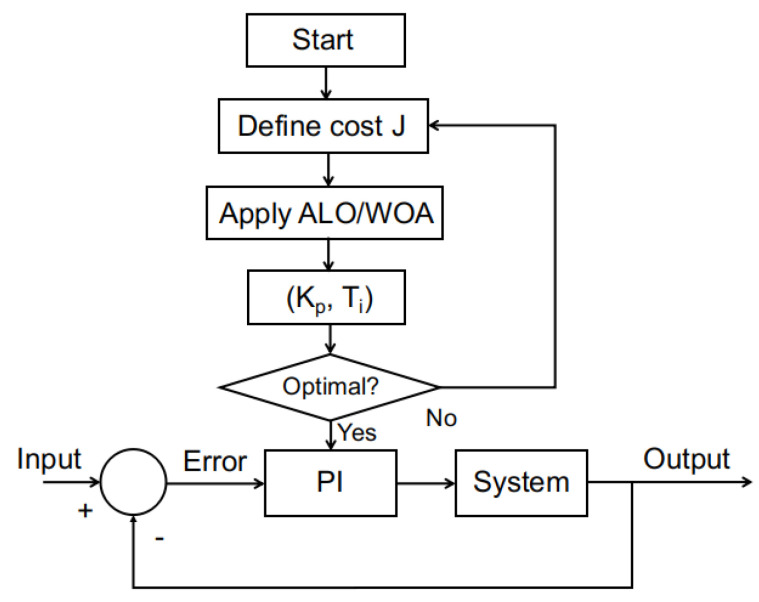
Flowchart of the methodology of PID tuning with ALO/WOA.

The methodology of the application of any of those metaheuristic optimization strategies to adjust the *PI* controller parameters is shown in the flowchart of Figure 5.

## 3. Discussion of the Results

The application of those two metaheuristic technique to the two selected systems has been carried out using a laptop computer equipped with an 4–core Intel(R) Core(TM) i5-3570K CPU at 3.40 GHz, a cache memory of 6144 kB and 12.0 GB of RAM, using the software Matlab R2023b. The corresponding results are shown and discussed in the next subsections.

### 3.1. PI Control of the Hoop and Ball System

**Figure 6:**
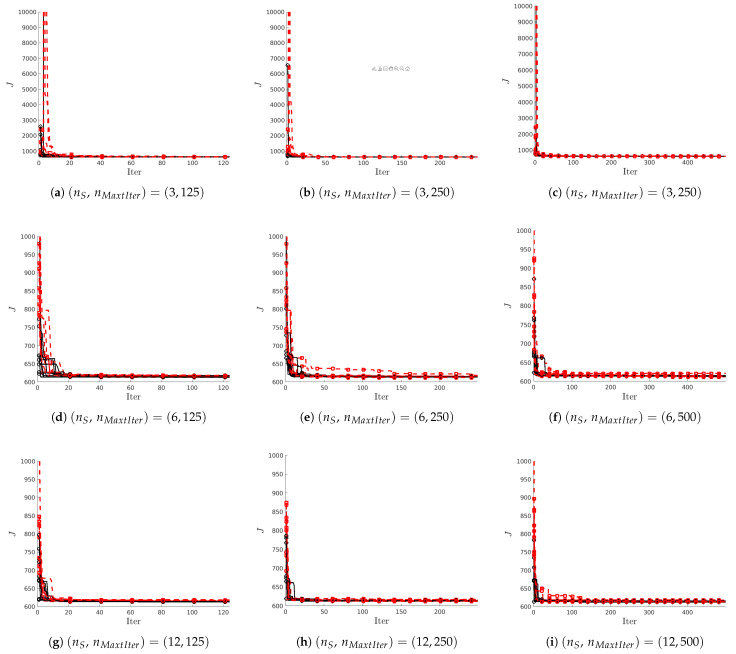
HB system: sensitivity analysis of convergence for different combinations of number of agents $n_{S}$ and number of iterations $n_{MaxtIter}$: $n_{R}=20$ runs for ALO (—∘—) and WOA (—□—), respectively.

In order to characterize the performance of both, the ALO and WOA techniques, for the tuning of *PI* controllers, we have carried out a sensibility analysis of the configuration parameters of those techniques, investigating the effects of the number of search agents $n_{S}$ and the number of iterations $n_{MaxIter}$ along the exploration the search space. For the hoop & ball system, we have considered $n_{S}\in \left[3,\;6,\;12\right]$ and $n_{MaxIter}\in \left[125,\;250,\;500\right]$. We have also taken into account the random nature of the algorithms; thus, we have carried out $n_{R}=20$ simulations for every combination $\left(n_{s},\;n_{MaxIter}\right)$ for both, ALO and WOA algorrithms. Note that any other configuration parameter is set as recommended in [15,16]. The results of these experiments are gathered in Table 1 and Table 2 and shown in Figure 6.

According to the data in Table 1 and Table 2, it is possible to see how –in general– the more agents exploring for longer time the search space, the smaller (the better) is on average the objective function *J*. However, there are some exceptions at: $\left(n_{S},\;n_{MaxIter}\right)=\left(12,\;250\right)$ *vs.*  $\left(n_{S},\;n_{MaxIter}\right)=\left(12,\;500\right)$ for ALO; this happens as well for WOA. Also for WOA and for the $n_{MaxIter}=500$ cases, note how doubling the number of search agents $n_{S}$ from 6 to 12 does not bring any significant improvement of the average value of the cost function *J*.

Also from Table 1 and Table 2, a practically linear scaling of the average computation time is observed both with the number of search agents $n_{S}$ and with the number of iterations $n_{MaxIter}$, for the ranges considered here.

The above analysis can be supplemented by inspection of Figure 6. The first observation is that the performance of ALO  is indistinguishable from that of WOA  on this problem. Moreover, quite often, solutions obtained with either method experience a significant decrease in the fitness function *J* at the beginning (in the first 20 iterations or so).

However, ALO solutions tend to decrease toward the minimum slightly faster than WOA solutions. In any case, and for this system, we observe how both techniques reach similar values for the cost function *J*. Note, however, that for some runs of the algorithm WOA , the final value of the cost *J* is slightly higher than both the other WOA  solutions and the ALO  solutions.

In view of the above results, further simulations have been performed using the combination $\left(n_{S},\;n_{IterMax}\right)=\left(6,\;500\right)$. Figure 2 shows a new set of results considering 10 new simulations (i.e., different seeds for the random number generator) for each metaheuristic optimization method.

The evolution of the cost function *J* (Figure 2a) is comparable to that shown in Figure 6. Figure 2b represents the solutions identified by ALO  or WOA  in the search space. It is evident that both methods tend to saturate the value of $K_{p}$. The values of $T_{i}$ have more variability, although they are mainly concentrated in the range $T_{i}\in \left[2,\;3\right]$. In Figure 2c,d, the outputs of the controlled system (θ(t) and y(t)) are shown for the different combinations of $\left(K_{p},\;T_{i}\right)$ identified by the ALO algorithm: in this case, the different solutions are quite close to each other. The same comments apply to the WOA  algorithm (Figure 2e,f). However, it should be noted that one of the solutions proposed by WOA  does not present overshoot. However, this solution does not correspond to the minimum *J*.

**Figure 7:**
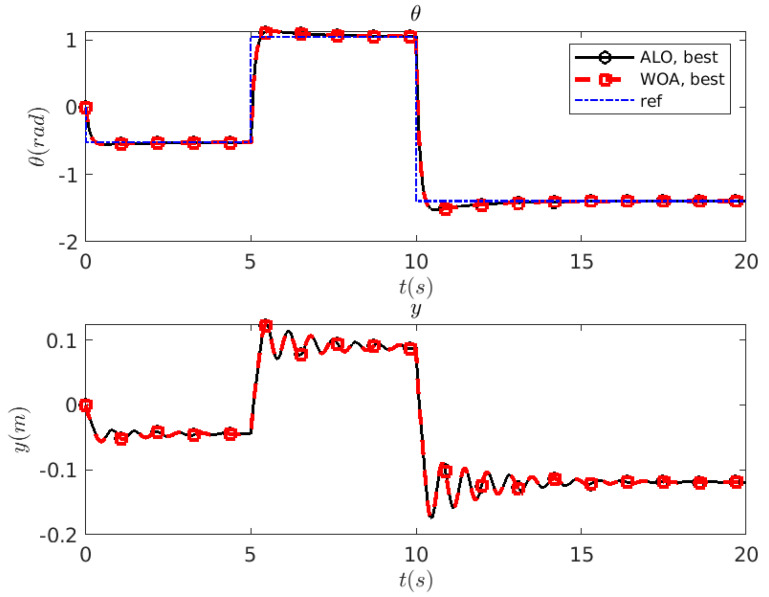
HB system: comparison of the optimal solutions identified by ALO (—∘—) and WOA (—□—).

The best solutions found by the algorithm ALO –are $\left(K_{p},\;T_{i}\right)=\left(3.099,\;2.122\right)$ with a cost value of J=611.788– and for WOA  the optimized gains are $\left(K_{p},\;T_{i}\right)=\left(3.100,\;2.121\right)$, with a cost value of J=612.012. These solutions are shown in Figure 7. Note how these solutions are visually indistinguishable.

**Figure 8:**
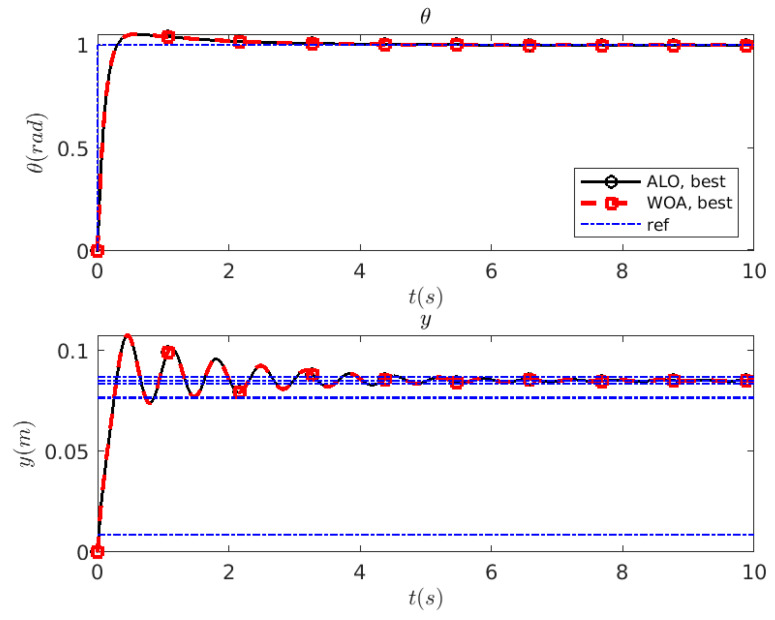
HB system: unit step response of the controlled system found by ALO (—∘—) and WOA (—□—).

The control solutions found by the optimization algorithms ALO/WOA  for this HB system can be compared against the unit step response of the same HB system reported in [13], which are found using an Adaptive Hybrid *PSO* strategy based on the squared error. The solutions presented in this work have faster response (i.e., rise time of y(t) of ≈0.23 s and settling time of ≈4.6 s vs., respectively, 0.84 s and 9.32 s in [13]) and a lower overshoot of 23.50% vs. 25.89% in [13], see Figure 8. Finally, it should be noted again that the other configuration parameters of the optimization algorithms are set as recommended in [15,16]. Therefore, the *PI* controller has been de facto tuned automatically, with no input information other than the allowed range in the space $\left(K_{p},\;T_{i}\right)$.

### 3.2. PI Control of Wind Turbine—Generator—Load System

In this subsection the results of the optimization of the *PI* controller for the angle of attack β of the WTGRL system with the two metaheuristic methods are presented and discussed.

The dynamic model of the WTGRL system is more complex than the one of the HB system. The WTGRL system includes several non–linear subsystems that interact together and are coupled. Additionally, the model uses powergui in a discretized setting, with a fairly high sampling frequency ($f_{s}=50\;$ kHz), which is necessary for stability considerations. Therefore, the computational cost of running this model even once is much larger. And many *runs* are necessary to perform a single optimization/tuning with either ALO or WOA algorithms. This situation prevents an exhaustive exploration of the performance with different parameter combinations $\left(n_{S},\;n_{MaxIter}\right)$, as shown for the HB system. We have nevertheless carried out a preliminary sensitivity analysis of the main configuration parameters of the optimization algorithms, $\left(n_{S},\;n_{MaxIter}\right)$, that may have a bigger influence in the behaviour of the optimization. The results are summarized in Table 3. Note how both algorithms, WOA/ALO , tend to saturate the gain $K_{p}$. The results also show a linear scaling trend with the number of iterations and the number of search agents.

**Figure 9:**
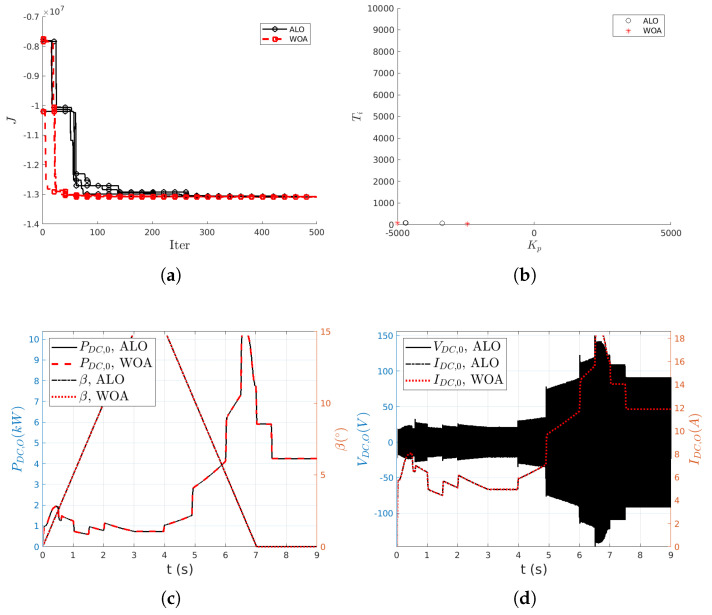
WTGRL system: in (**a**), evolution of the cost function; in (**b**), different solutions identified by ALO (∘) and WOA (*); in (**c**,**d**), comparison of the optimal solutions found by ALO and WOA  algorithms.

Next, a more exhaustive exploration of the space $\left(K_{p}\;T_{i}\right)$ has been performed, considering $\left(n_{S},\;n_{MaxIter}\right)=\left(6,\;500\right)$ and three simulations for each metaheuristic optimization technique. The results of this study are summarized in Table 4 and shown in Figure 9.

Given the data in Table 4, both ALO  and WOA  find a similar value for the cost function *J* on average. It can also be observed, as previously mentioned, the significant increase in the computation time required for an individual simulation, which goes from tens of minutes to more than a day.

Figure 9a shows the evolution of the fitness function *J* with the number of iterations for the WTGRL system. It is possible to see a significant decrease of *J* at the beginning. However, in this case, around 150 iterations are necessary to get close to the optimal value, and still appreciable decreases are observed at iteration ≈350. Contrary to what is observed in Figure 2a for the HB system, in these cases the solutions of WOA  tend to decrease faster than those of the ALO  algorithm. Again, it is observed how both techniques reach similar values of the cost function *J*.

Figure 9b shows the location of the solutions found by the ALO or WOA  algorithms in the search space. In this case, it is possible to see how both optimization algorithms, ALO and WOA , tend to saturate the value of $K_{p}$ at 2 after 3 iterations. The values of $T_{i}$ show again more variability.

More specifically, for the WTGRL system, the optimal values of the gains of the controller found are: $J=-1.3086633\times 10^{7}$ with tuning parameters $\left(K_{p},\;T_{i}\right)=\left(4707.6,\;85.6\right)$, with the ALO algorithm; and $J=-1.30866328\times 10^{7}$ with values of the controller gains $\left(K_{p},\;T_{i}\right)=\left(2440.9,\;44.2\right)$, with the WOA algorithm. The value of the cost function is very similar for the two techniques, although the controller parameters are radically different: both the ratio $K_{p,ALO}/K_{p,WOA}$ and $T_{i,ALO}/T_{i,WOA}$ are ≈2. However, inspection of Figure 9c,d reveals that, regardless of the controller used, the solutions with respect to the evolution of the angle of attack, the dissipated power, the voltage and the current intensity in the load overlap for both methods.

It should be noted that, for greater clarity in the visualization of the performance of the algorithms, only the ALO  voltage is shown in Figure 9d since the results can be considered equal for both techniques. The voltage obtained with the parameters found by WOA  is superimposed on the ALO  result.

The overlapped solutions shown in Figure 9c,d are consistent with the well known fact that equivalent behaviors of a controlled system can be obtained for *PI* controllers with different values of its parameters.

In this sense, it is possible to affirm the ability of the ALO  and WOA  techniques to find different combinations of controller tuning parameters that lead to an optimized response of the complex and nonlinear WTGRL  system. The rest of the configuration parameters of the ALO/WOA  algorithms have been set to the value recommended in [15,16], and, therefore, it has only been necessary to know the desired range in the $\left(K_{p},\;T_{i}\right)$ space to find the optimal *PI*  controller.

## 4. Conclusions and Future Works

In this work, the performance of two bio-inspired metaheuristic optimization algorithms, the Antlion Optimizer (or ALO) and the Whale Optimization Algorithm (or WOA), has been investigated. They have been applied to the adjustment of a conventional Proportional Integral (PI) controller for two systems of different complexity, the electromechanical Hoop and Ball (HB) system, and a wind turbine, generator, rectifier and load (WTGRL) system. Both techniques have been shown to present a robust and effective performance in the problems addressed.

The application to these systems is motivated by comparing both algorithms in two different scenarios. The Hoop and Ball (HB) system, allows a linearized description of it, while the wind turbine (WTGRL) system is an example of a complex non-linear dynamic system, of interest in the field of renewable energies, which cannot be adequately represented by a linear model.

In evaluating the performance of the ALO and WOA techniques for tuning the *PI*  controllers, a sensitivity analysis of the two most relevant parameters has been considered: the number of agents $n_{S}$ and the maximum number of iterations $n_{MaxIter}$. The repeatability properties (i.e., the dependence on the seed for the random number generator) and the computational effort required have also been studied.

From the results obtained with both optimization techniques, it has been observed that both methods reach similar optimal values for the fitness (cost) function *J*. In general, the more agents and the more time they are allowed to explore the search space, the better solution, i.e., the lower the value of *J*, is found.

Regarding the computational time, it is observed that $t_{Opt}$ increases linearly with both the number of agents $n_{S}$ and the number of iterations $n_{MaxIter}$ with both optimization techniques.

On the other hand, the versatility of these techniques is shown since in some cases the ALO and/or WOA  techniques have arrived at very different solutions, finding very different values of the parameters $K_{p}$/$T_{i}$ of the controller that, however, give the same fitness function and very similar responses of the dynamic systems considered.

In general, the effectiveness and robustness demonstrated by both bio-inspired metaheuristic techniques, the Antlion Optimizer and the Whale Optimization Algorithm, shown in their application to various optimization problems, have been confirmed here for the tuning of a conventional controller for non-linear dynamical systems.

Another interesting consideration that can be deduced is that ALO/WOA methods should be used, whenever computationally affordable, by considering a sufficient number of runs, i.e., $n_{R}$. This allows for considering the randomness of the initial conditions and the repeatability of the experiments and results obtained.

This last conclusion supports the need to have relatively simple models in terms of their simulation. This is one of the main limitations identified in the application of these techniques. Indeed, the faster a model is executed (the better the computational efficiency), the greater the number of search agents, iterations and realizations that can be performed and, therefore, the more complete the exploration of the solution space will be.

Other future work includes the possibility of applying these techniques ALO/WOA to other control problems in various fields. Specifically, and addressing another limitation of these metaheuristic methods, it would be desirable to have well-defined problems where these or other techniques could be compared. For this reason, in this work an effort has been made to establish the repeatability of the experiments.

## Figures and Tables

**Table 1:** HB system: optimal minimum reached $\bar{J}$ and computational time needed ${\bar{t}}_{Opt}$ (in seconds) for the ALO technique, averaged over $n_{R}=20$ runs.

			$n_{\mathrm{MaxIter}}=125$			$n_{\mathrm{MaxIter}}=250$			$n_{\mathrm{MaxIter}}=500$	
		$\bar{J}$		${\bar{t}}_{Opt}\;\left[s\right]$	$\bar{J}$		${\bar{t}}_{Opt}\;\left[s\right]$	$\bar{J}$		${\bar{t}}_{Opt}\;\left[s\right]$
	$n_{S}=3$	617.4049		103.3	614.8049		205.8	614.4621		411.8
	$n_{S}=6$	614.9633		206.0	614.4436		411.7	614.0940		825.1
	$n_{S}=12$	613.8919		413.5	613.4070		825.0	613.9141		1650.6

**Table 2:** HB system: optimal minimum reached $\bar{J}$ and computational time needed ${\bar{t}}_{Opt}$ for the WOA technique, averaged over $n_{R}=20$ runs.

			$n_{\mathrm{MaxIter}}=125$			$n_{\mathrm{MaxIter}}=250$			$n_{\mathrm{MaxIter}}=500$	
		$\bar{J}$		${\bar{t}}_{Opt}\;\left[s\right]$	$\bar{J}$		${\bar{t}}_{Opt}\;\left[s\right]$	$\bar{J}$		${\bar{t}}_{Opt}\;\left[s\right]$
	$n_{S}=3$	618.9770		102.6	614.9905		205.1	615.8912		411.1
	$n_{S}=6$	615.6602		205.4	614.8341		411.4	614.3943		825.7
	$n_{S}=12$	615.2329		413.2	613.8167		826.7	614.2489		1652.4

**Table 3:** WTGRL system, initial configuration study: computational time ${\bar{t}}_{Opt}$ (in hours) and solution found $\left(K_{p},\;T_{i}\right)$ for both, the ALO and the WOA techniques, averaged over $n_{R}=3$ runs.

	$\left(n_{S},\;n_{\mathrm{MaxIter}}\right)$	$t_{\mathrm{Opt}}$(h)	$K_{p}$	$T_{i}$
	(6,50)	4.1	−4991.1	9214.9
ALO	(6,100)	9.2	−4993.5	91.27
	(12,50)	9.1	−870.3702	54,827
	(6,50)	4.9	−5000	578.99
WOA	(6,100)	10.2	−4109.7	75.14
	(12,50)	9.3	3147.2	57,430

**Table 4:** WTGRL system: optimal minimum reached $\bar{J}$ and computational time needed ${\bar{t}}_{Opt}$ (in hours) for both the ALO and the WOA  techniques, averaged over $n_{R}=3$ runs.

			ALO			WOA	
		$\bar{J}$		${\bar{t}}_{Opt}\;\left[h\right]$	$\bar{J}$		${\bar{t}}_{Opt}\;\left[h\right]$
		$-1.308182\times 10^{7}$		29.1	$-1.308423\times 10^{7}$		28.76

## Data Availability

Data generated during the current study are available from the corresponding authors on reasonable request.

## Abbreviations

The following abbreviations are used in this manuscript:

ALO	Antlion Optimization
WOA	Whale Optimization Algorithm
HB	Hoop & Ball
WTGRL	Wind turbine–generator–rectifier–load
*PI*	Proportional Integral
*PID*	Proportional Integral Derivative
MPPT	Maximum Power Point Tracking
*GA*	Genetic Algorithm
*PSO*	Particle Swarm Optimization
